# The physiological strain index does not reliably identify individuals at risk of reaching a thermal tolerance limit

**DOI:** 10.1007/s00421-021-04642-3

**Published:** 2021-03-07

**Authors:** Sarah L. Davey, Victoria Downie, Katy Griggs, George Havenith

**Affiliations:** 1 Environmental Ergonomics Research Centre, Loughborough Design School, Loughborough University, Loughborough, LE11 3TU UK; 2 Department of Health and Life Sciences, Coventry University, Oxford, UK; 3 English Institute of Sport, Manchester, UK; 4 Department of Engineering, School of Science and Technology, Nottingham Trent University, Nottingham, UK

**Keywords:** Physiological strain index, Heat stress, Heat stress indices, Thermal tolerance limit, Heat illness, Hyperthermia-induced fatigue

## Abstract

**Purpose:**

The physiological strain index (PSI) was developed to assess individuals’ heat strain, yet evidence supporting its use to identify individuals at potential risk of reaching a thermal tolerance limit (TTL) is limited. The aim of this study was to assess whether PSI can identify individuals at risk of reaching a TTL.

**Methods:**

Fifteen females and 21 males undertook a total of 136 trials, each consisting of two 40–60 minute periods of treadmill walking separated by ~ 15 minutes rest, wearing permeable or impermeable clothing, in a range of climatic conditions. Heart rate (HR), skin temperature (*T*_sk_), rectal temperature (*T*_re_), temperature sensation (TS) and thermal comfort (TC) were measured throughout. Various forms of the PSI-index were assessed including the original PSI, PSI_fixed_, adaptive-PSI (aPSI) and a version comprised of a measure of heat storage (PSI_HS_). Final physiological and PSI values and their rate of change (ROC) over a trial and in the last 10 minutes of a trial were compared between trials completed (C, 101 trials) and those terminated prematurely (TTL, 35 trials).

**Results:**

Final PSI_original_, PSI_fixed_, aPSI, PSI_HS_ did not differ between TTL and C (*p* > 0.05). However, differences between TTL and C occurred in final *T*_sk_, *T*_re_–*T*_sk_, TS, TC and ROC in PSI_fixed_, *T*_re_, *T*_sk_ and HR (*p* < 0.05).

**Conclusion:**

These results suggest the PSI, in the various forms, does not reliably identify individuals at imminent risk of reaching their TTL and its validity as a physiological safety index is therefore questionable. However, a physiological-perceptual strain index may provide a more valid measure.

## Introduction

Heat stress experienced within the workplace can result in hyperthermia-induced fatigue (Nybo et al. 2014), which, left untreated or not identified early, can develop into more serious heat-related illnesses such as heat exhaustion, heat syncope, or in extreme cases heat stroke and death (Arbury et al. 2014). Hyperthermia-induced fatigue (HIF) as well as heat-related illnesses (both of which can be considered to cause an individual to reach a thermal tolerance limit) are of a major concern for industries as they can lead to accidents, absenteeism and can negatively affect the health and safety of their workers (Flouris et al. 2018; Seppänen and Fisk 2005). It can also lead to a reduction in work productivity (Flouris et al. 2018; Foster et al. 2019); an issue detrimental to any industry.

Workers who are required to wear personal protective equipment (PPE) or, due to working outdoors, are exposed to high levels of heat, humidity and/or solar radiation are considered to be most at risk of HIF and heat-related illnesses (ILO 2016; Schulte et al. 2016). To combat this issue, several heat strain indices or monitoring tools have been developed to inform occupational heat stress standards or guidelines (Havenith and Fiala 2015). These guidelines generally provide recommended heat exposure limits based on environmental (e.g. ambient temperature, relative humidity, wind speed, clothing insulation) or physiological (core temperature, heart rate, skin temperature) parameters, or a combination of both, to ensure that the group’s average core temperature (*T*_c_) does not exceed 38.0 °C (ACGIH 2009; Jendritzky et al. 2012; National Institute for Occupational Safety and Health [NIOSH] 2016; ISO 2004; Malchaire et al. 2001). The main criticism of these indices, however, is that they are based on average group responses, are therefore conservative, and not appropriate to measure detrimental levels of heat stress at an individual level.

The physiological strain index (PSI) was developed to reduce incidences of heat-related illnesses at an individual level (Moran et al. 1998b). Unlike the predictive standards mentioned above, the PSI is a ‘live’ monitoring tool and is calculated using measures of rectal temperature (*T*_re_) and heart rate (HR) to reflect the combined strain of the cardiovascular and thermoregulatory systems; with both parameters contributing equally in evaluating physiological strain. Physiological strain is described on a universal scale of 0–10 and is used to classify individuals into certain risk categories, with 0 representing no physiological strain, 10 representing highest physiological strain and ≥ 7.5 being considered as high risk for thermal injury (Buller et al. 2008; Moran et al. 1998b). While PSI was in part developed as a heat illness prevention tool, it has mainly been validated regarding its ability to distinguish between different levels of thermal strain in different heat stress scenarios including both hot-dry and hot-wet environments, differing hydration levels, impact of differing PPE and between the sexes (Moran et al. 1998a, 1999; Moran 2000; Petruzzello et al. 2009). These validation studies confirm the close relationship between environmental heat stress and the cardiovascular (i.e. heart rate) and thermoregulatory systems (i.e. core temperature) and, consequently, the ability of PSI to discriminate physiological strain in response to differing levels of heat stress, or in situations where thermoregulation is impaired. However, as the data generally reported in these studies are on individuals who tolerated the different heat exposures, there is limited evidence supporting the use of PSI to identify individuals at potential risk of HIF or heat-related illnesses, or in other words, reaching a thermal tolerance limit.

Exhaustion or fatigue associated with an elevated *T*_c_ (i.e. HIF) is becoming more widely recognised as an event caused by the interplay of both central and peripheral physiological factors, alongside psychological processes such as motivation, previous experience and expectation of demand (Flouris and Schlader 2015; Nybo et al. 2014). The multi-factorial nature of HIF is evidenced by the observation that the *T*_c_ at which the onset of HIF occurs is highly individualised (Ely et al. 2009). Other parameters have been associated with HIF, such as cardiovascular strain reflected in HR (Périard et al. 2011), supporting the notion that PSI could be used to identify individuals at risk of HIF. However, to the authors’ knowledge this has yet to be explored. Therefore, the aim of this study was to assess whether PSI can identify individuals at risk of HIF and/or heat-related illnesses (i.e. individuals reaching a thermal tolerance limit) in a variety of heat stress scenarios designed to represent conditions experienced in both indoor and outdoor worksites. The chosen heat stress scenarios varied in regard to climatic conditions [i.e. ambient temperature (25–40 °C), relative humidity (20–85%), and the presence of simulated solar radiation], the clothing worn (i.e. clothing of different vapour permeability) and the type and length of metabolic activity.

## Methods

### Participants

Fifteen females and 21 males participated in the study (Females: age = 25.2 ± 6.7 years; body mass = 61.3 ± 5.9 kg; peak oxygen uptake = 47.45 ± 11.09 ml kg min^−1^; body fat = 20.34 ± 6.11%, Males: age = 24.8 ± 5.7 years; body mass = 76.1 ± 9.5 kg; peak oxygen uptake = 50.69 ± 9.34 ml kg min^−1^; body fat = 14.02 ± 5.9%). All participants were verbally briefed, issued with a participant information sheet, and gave written informed consent. Ethical approval for the procedures was obtained from Loughborough University ethics committee and designed in accordance with the 2013 Declaration of Helsinki regarding human experimentation.

### Experimental procedures

Prior to commencing the main trials, all participants underwent a maximal intensity fitness test on a treadmill to determine peak oxygen uptake ($\dot{V}O_{{2{\text{peak}}}}$). Participants were also measured for height, body mass and estimated body fat percentage (Durnin and Womersley 1974). Participants undertook between two and nine trials (each separated by at least 3 days). Trials were completed at the same time of day for each participant (either 8 am, 12 pm or 4 pm) to account for circadian rhythm changes in core temperature and cardiovascular responses.

**Fig. 1:**
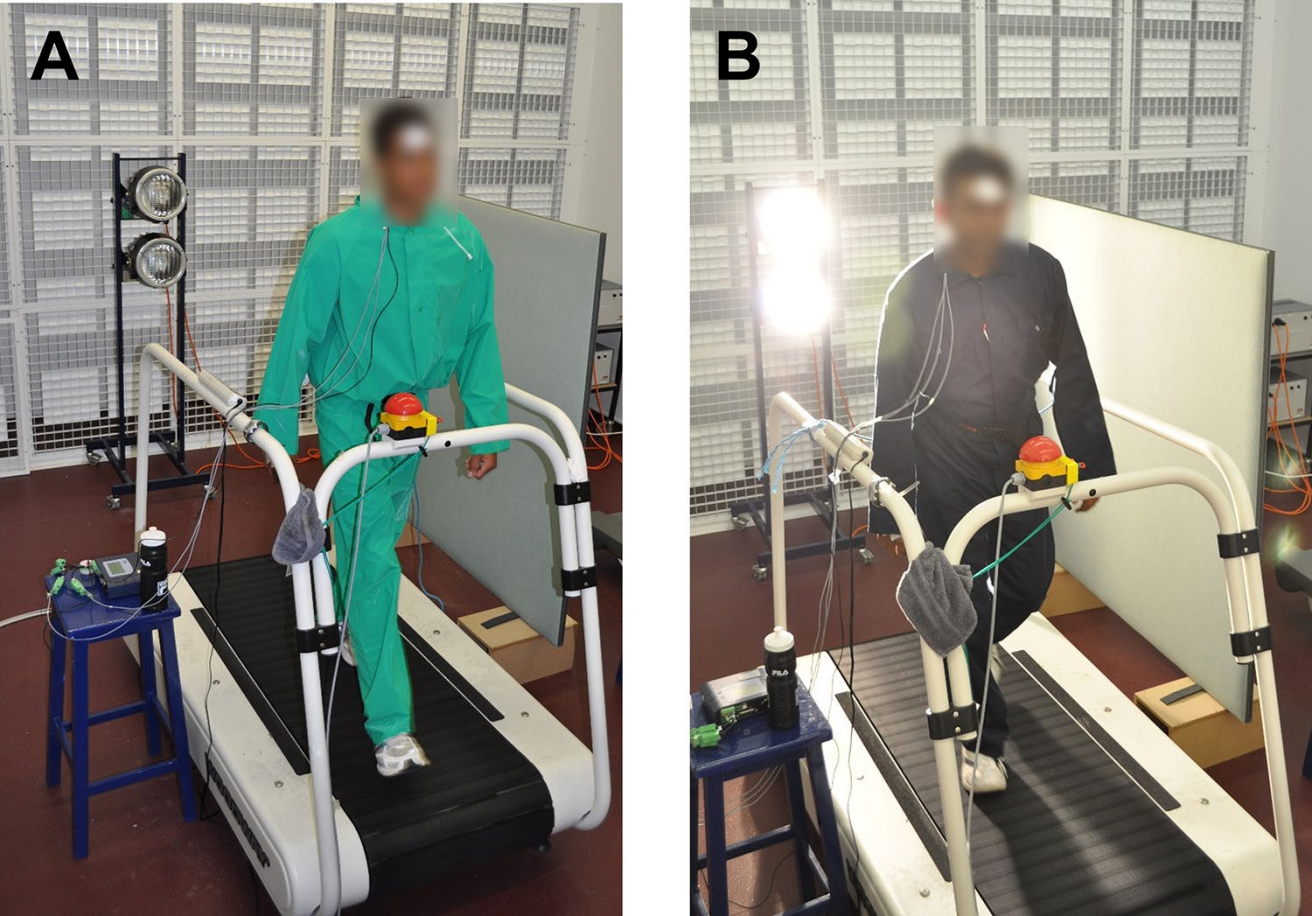
Two of the types of protective clothing worn by the participants: **a** impermeable clothing and **b** permeable clothing, plus localised thermal radiation directed onto the back of the participants

Each trial differed either in ambient temperature and relative humidity (rh), the type of clothing worn (impermeable or permeable), the presence of a radiant heat source (~ 530 Wm^−2^ focused on the back of the participant) and/or the work/rest regime (see Table 2 and Fig. 1). The rationale behind the wide variation in conditions (work and climate) of the trials was to ensure the results derived would be relevant to the wide variation of conditions encountered in real life work scenarios (e.g. indoor and outdoor), rather than to a single heat/work stress condition. The chosen conditions of the trials were also aimed at participants achieving body core temperatures above 38.5 °C in all trials.

Each trial started with a 10-min seated rest period to allow for a stabilisation in skin and rectal temperatures. With the exception of condition 3, trials in conditions 1–9 consisted of completing a work/rest regime that involved two 40- to 60-min periods of walking on a treadmill at an exercise intensity of ~ 40% $\dot{V}O_{{2{\text{peak}}}}$ separated by seated rest in ~ 22 °C, 50% rh outside the climatic chamber. The duration of seated rest was determined by the rate of recovery/decline in rectal temperature (*T*_re_) of the individual, with participants returning to the climatic chamber once *T*_re_ had dropped by 0.4 °C (average time = 10–20 min).

Condition 3 was designed to simulate a typical work regime of an industrial worker and to evaluate various transitions of metabolic activity and, therefore, heat production. The work/rest regime of trials in this condition involved two 25-min periods of treadmill walking of varying intensity involving 5 min of moderate exercise (~ 35% $\dot{V}O_{{2{\text{peak}}}}$), 5 min of moderate to hard exercise (~ 65% $\dot{V}O_{{2{\text{peak}}}}$) and 15 min of moderate exercise (~ 35% $\dot{V}O_{{2{\text{peak}}}}$) separated by 10 min of seated rest in the climatic chamber. After the completion of the second 25-min period of treadmill walking, the participant rested in ~ 22 °C, 50% rh outside the climatic chamber (as per conditions 1–9), before completing another 25-min period of treadmill walking of varying intensity (thus completing a total of 3 × 25 min periods of treadmill walking) followed by 10 min of seated rest in the climatic chamber as part of the same trial. Participants were allowed to drink ad libitum throughout a trial. To reflect a typical working scenario where hydration practices would be encouraged, but not enforced, participants were encouraged to be euhydrated (i.e. urine to be pale yellow in colour) at the beginning of a trial, but hydration status was not measured. To assess the level of hypohydration in participants over a trial the difference between pre and post nude body mass was measured. The amount of fluid consumed and urine volume was also measured to assess sweat rate.

Two 1000 W Metal Halide Compact Source Iodide (CSI) lamps (GE Lighting) were used to simulate solar radiation. These lamps produce light with a spectrum similar to that of sunlight. The radiation was directed from behind the participant and angled to concentrate the radiation onto the posterior torso region. The intensity and direction of the radiation were controlled by the distance (~ 2.3 m) and angle of the lamps from the participant walking on the treadmill. The amount of direct radiation was measured with a Pyranometer (CM11, Kipp & Zonen, Netherlands) prior to and every 10 min during the trial.

The participants wore either a permeable or impermeable two layered clothing system, matched for dry insulation. Each clothing system included a long-sleeved shirt (100% cotton) and shorts (100% Lycra) as the first layer, then either the permeable or impermeable all-in-one suits as the second layer, worn with a belt at the waist. The total thermal resistance and evaporative resistance for the permeable clothing system were 0.166 m^2^ K W^−1^ and 42.4 m^2^ Pa W^−1^ and for the impermeable clothing system 0.167 m^2^ K W^−1^ and 213.3 m^2^ Pa W^−1^, respectively.

In the present study, a trial could be stopped prematurely either by the researcher or volitionally by the participant. Reasons for terminating the trial included the following: the participant feeling excessively fatigued or exhibiting signs and symptoms of heat-related illnesses, e.g. mental confusion, lack of co-ordination, clammy, pale skin, nausea, dizziness and headache. All researchers were experienced in administering exercise protocols in both thermally challenging and thermoneutral conditions and therefore able to recognise signs and symptoms of heat-related illnesses and HIF. A trial terminated prematurely was assigned to group TTL (thermal tolerance limit) to represent a population who could not complete the heat stress scenario due to HIF and/or other symptoms of heat-related illnesses. For simplicity the term of thermal tolerance limit will be used hereafter to describe incidences of HIF and/or other symptoms of heat-related illnesses. Trials that were completed were assigned to group C (Control). To ensure the safety of participants, the following criteria were also used for stopping a trial (1) *T*_re_ > 39.5 °C; (2) HR above 95% of age-predicted maximum. Only 4 trials were ceased prematurely due to the latter safety criteria. As the participants in these trials did not display any signs and symptoms of a heat-related illness or reported feelings of fatigue, the data from these trials were excluded from the analyses. The rationale for this exclusion was the uncertainty to whether the participant would have gone on to complete the trial or stop prematurely due to reaching a thermal tolerance limit.

### Measurements

The following measurements were recorded in all trials: (1) *T*_re_ (self-inserted; Edale Instruments, Cambridge, UK); (2) local skin temperature of the forehead (*T*_head_), chest (*T*_chest_), upper back (*T*_u.back_), upper arm (*T*_u.arm_), lower arm (*T*_l.arm_), hand (*T*_hand_), lower back (*T*_l.back_), abdomen (*T*_abdominal_), thigh (*T*_thigh_), calf (*T*_calf_) and foot (*T*_foot_) were measured on the right-hand side of the body (iButtons thermochrons, Homechip, Milton Keynes, UK) and the average of these temperatures was used to calculate mean skin temperature (*T*_sk_); (3) HR (RS 800, Polar, Finland). Environmental measurements of ambient temperature, relative humidity, radiant heat and wind speed were also recorded (Testo 453, Testo SE & Co, Germany). Every five minutes, participants were asked for their subjective rating of thermal comfort (TC) and temperature sensation (TS). Both the TC and TS were recorded on horizontal visual analogue scales ranging from very comfortable to very uncomfortable and very cold to very hot, respectively. The corresponding scores ranged from 0 to 20 with higher scores representing feeling more uncomfortable on the TC scale and feeling hotter on the TS scale (Davey et al. 2007).

### Calculations

#### Physiological strain index (PSI)

Several variations to the calculation of PSI were assessed as follows:

(1) PSI_original_ (Moran et al. 1998a, b): PSI calculated using the actual resting values for *T*_re_(0) and HR(0):

$${\text{PSI}} = 5 \times \frac{{T_{{{\text{re}}}} (t) {-} T_{{{\text{re}}}} (0)}}{{39.5 {-} T_{{{\text{re}}}} (0)}} + 5 \times \frac{{{\text{HR}}(t) {-} {\text{HR}}(0)}}{{180 {-} {\text{HR}}(0)}},$$

where *T*_re_(*t*) = current *T*_re_, *T*_re_(0) = initial *T*_re_, HR(*t*) = current HR and HR(0) = initial HR.

(2) PSI_fixed_ (Buller et al. 2008): PSI calculated using fixed values for the starting point, i.e. *T*_re_(0) and HR(0) at 37.0 °C and 70 beats min^−1^, respectively. This version of the PSI was assessed as, on a practical level, it is not always possible to establish resting values in a thermoneutral environment. The fixed values used are similar to the mean initial *T*_re_ and HR values recorded at the beginning of all heat exposures, i.e. 37.17 (± 0.26) °C and 76 (± 13) beats min^−1^:

$${\text{PSI}}_{{{\text{fixed}}}} = 5 \times \frac{{T_{{{\text{re}}}} (t) {-} 37.0}}{{39.5 {-} 37.0}} + 5 \times \frac{{{\text{HR}}(t) {-} 70}}{{180 {-} 70}}$$

(3) The adaptive-PSI (aPSI) (Buller et al. 2016) was also assessed as it has been shown to better identify levels of heat strain than the PSI_fixed_ (Buller et al. 2016). The aPSI adjusts the critical core temperature of 39.5 °C used in PSI_fixed_ by the gradient between *T*_c_ and *T*_sk_as follows:

$${\text{PSI}} = 5 \times \frac{{T_{{{\text{re}}}} (t) {-} T_{{{\text{re}}}} (0)}}{{39.5 + \frac{{\left[ {T_{{{\text{re}}}} \left( t \right) - T_{{{\text{sk}}}} \left( t \right)} \right] - 4}}{4} {-} T_{{{\text{re}}}} (0)}} + 5 \times \frac{{{\text{HR}}(t) {-} {\text{HR}}(0)}}{{180 {-} {\text{HR}}(0)}}$$

(4) Finally, to determine whether incorporating skin temperature in the form of heat storage into the PSI equation would provide a better prediction of an individual reaching a thermal tolerance limit, the following modification to the calculation of PSI was also evaluated:

$${\text{PSI}}_{{{\text{HS}}}} = 5 \times \frac{{{\text{HS}}(t)}}{{{\text{HS}}_{{{\text{crit}}}} {-} {\text{HS}}\left( 0 \right)}} + 5 \times \frac{{{\text{HR}}(t) {-} {\text{HR}}(0)}}{{180 {-} {\text{HR}}(0)}},$$

where HS(*t*) = (0.8 • [*T*_re_(*t*) − *T*_re_ (0)]) + (0.2 • [*T*_sk_(*t*) − *T*_sk_(0)]) • 3.49 (J g^−1^); Havenith et al. (1995), HS_crit_ = (0.8 • [39.5 − 37.0]) + (0.2 • [37.5 − 32.5]) • (J g^−1^); HS (0) = 0.

#### Rate of change in PSI, ***T***_sk_, HR and ***T***_re_

As group TTL experienced shorter heat exposures than group C (see “Results” section) it is plausible that they would have reached a certain PSI earlier than group C so, as a consequence, the rate of change (ROC) in PSI_fixed_ was also calculated using the following equation:

$${\text{ROC in PSI}}_{{{\text{fixed}}}} = \frac{{{\text{Final PSI}}_{{{\text{fixed}}}} - {\text{Initial PSI}}_{{{\text{fixed}}}} }}{{\text{Exposure time (s)}}}$$

Across a trial the ROC in *T*_re_, *T*_sk_, *T*_re_–*T*_sk_ and HR were calculated in the same manner as ROC PSI_fixed_. The ROC in the last ten minutes of a trial was also calculated (i.e. *T*_re10_, *T*_sk10_, HR_10_, *T*_re10_–*T*_sk10_ and PSI_fixed10_)_._ The latter parameters were included as they were considered to be more pragmatic to incorporate into a heat strain index/heat stress monitor as generally the length of exposure before reaching a thermal tolerance limit is generally unknown, especially on an individual basis.

### Statistical analysis

All data are presented as means ± standard deviation (SD) unless stated otherwise. Cumulative frequency graphs were used to assess the validity of the PSI_fixed_ and the ROC in PSI_fixed_ to identify individuals at risk of reaching a thermal tolerance limit. To determine whether PSI can identify individuals reaching a TTL, regardless of the type of heat stress scenario i.e. condition, independent *t*-tests (or where variables were not normally distributed the Mann–Whitney *U* test) were used to compare variables recorded at the end of a trial between groups TTL and C. These measures included final PSI_original_, PSI_fixed_, aPSI, PSI_HS,_ HR, *T*_re_, *T*_sk_, *T*_re_–*T*_sk_, TS, TC, ∆ HR, ∆ *T*_re_, ∆ *T*_sk_, ∆ *T*_re_, ROC in PSI_fixed_, *T*_re_, *T*_sk_, HR, *T*_re_–*T*_sk_ (both over a trial and in the last 10 min of a trial). To determine whether there was a condition effect, only the conditions that produced the most cases of individuals reaching a TTL (i.e. conditions, 1, 2 and 6), were used to perform a two-way independent measures ANOVA to assess the interaction of group and condition on PSI_fixed_ and ROC in PSI_fixed_ (both over a trial and in the last 10 min of a trial). The ANOVA was only performed using the 3 conditions listed due to the variance in the number of participants undertaking and the number of participants reaching TTL in the other conditions (Table 4). When a significant difference was found for a main effect (group or condition), post-hoc pair-wise comparisons were made incorporating a Bonferroni adjustment. Effect sizes for pairwise comparisons were calculated using either corrected Cohen’s *d* (i.e. hedges’s *g*; Cumming 2012) for independent *t*-tests or the conversion of *z* scores to *r* values for Mann–Whitney *U* tests (Field, 2013). Commonly used interpretations of cohen’s *d* and *r* vales is to refer effect sizes as small (*d* ≥ 0.2, *r* = 0.1–0.3), medium (*d* ≥ 0.5, *r* = 0.3–0.5) and large (*d* ≥ 0.8, *r* = 0.5–1.0) (Cohen 1988). All statistical procedures were performed using the Statistical Package for the Social Sciences 24.0 for Windows (SPSS, Inc., Chicago, IL, USA). Statistical significance was set at *p* < 0.05.

## Results

Out of 150 trials, 115 trials (74.8%) were completed and 35 trials (22.7%) were stopped prematurely due to the participant reaching a thermal tolerance limit. Every participant who participated in condition 7 completed the trial; therefore, this condition was removed from the analyses. However, a description of this condition and corresponding PSI values have been included in Tables 2, 4, to provide an example of a heat stress scenario where individuals reaching a thermal tolerance limit is less likely to occur, or, in other words, a heat stress scenario tolerated by the majority and therefore maybe deemed less of a health and safety risk. Consequently, the 101 trials that were completed comprised group C and the 35 trials being stopped prematurely comprised group TTL.

The average length of heat exposure for group TTL was 87.74 ± 29.26 min (range 35–125 min) and 118.31 ± 9.14 min (range 100–145 min) for group C. Table 1 demonstrates that the physical characteristics of participants were similar between groups TTL and C. Most cases of TTL occurred in conditions 1, 2 and 6; conditions that generally had the highest ambient water vapour pressure or included impermeable clothing, both of which would have induced a higher water vapour at the skin surface compromising evaporative heat loss (see Tables 2, 4). The majority of participants completed at least 80% of their trials (median = 80%) with only two participants unable to complete any of the trials attempted (no of trials attempted = 2–3). In all trials attempted, these two participants ceased exercising due to experiencing either fatigue, dizziness or a headache. These two participants’ final *T*_re_ and HR ranged between 38.0 and 38.7 °C and 130–150 beats min^−1^, respectively which could be considered moderate thermal strain. Across all conditions the % change in nude body mass was − 0.42 ± 0.81%. This level of hypohydration experienced during the trials is equivalent to those likely experienced in workplaces where participants have access to fluids and are educated on the importance of hydration (Brearley et al. 2015).

**Table 1:** Physical characteristics of the participants in groups C and TTL; mean ± SD (*n* = 36)

Physical characteristics	*C*	TTL
*N*	13	23
Age (years)	25.8 ± 7.1	25.3 ± 5.5
Body mass (kg)	69.7 ± 12.5	70.9 ± 10.0
Body surface area (m^2^)	1.84 ± 0.21	1.85 ± 0.16
Body fat (%)	14.66 ± 7.49	17.50 ± 7.23
Peak oxygen uptake (ml kg min^−1^)	52.42 ± 9.90	47.26 ± 10.15

None of the variables were significantly different between groups. TTL = Group comprised of participants who stopped a trial prematurely at least once due to hyperthermia-induced fatigue or another heat-related symptom (i.e. reached a thermal tolerance limit), *C* = Group comprised of participants who completed all the trials they attempted

**Table 2:** Environmental conditions, clothing worn, work pattern and representative heat stress scenario for each of the nine conditions

Condition	Ambient temperature (°C)	Relative humidity (%)	WBGT (°C)	WVP (g m^−3^)	Solar radiation	Clothing	Work pattern ǂ	Heat stress scenario
1	40	35	31.85	17.90	No	Permeable	60 min moderate exercise, rest* (~ 22 °C, 50% rh) until a 0.4 °C fall in rectal temperature, 40 min moderate exercise	Indoors in a hot factory. Basic level PPE
2	33	85	30.12	30.45	No	Permeable	Same as condition 1	Indoors in a hot, humid environment. Basic level PPE
3	40	35	31.85	17.90	No	Permeable	2 × work cycle** seperated by 10 min seated rest in chamber, rest* (~ 22 °C, 50% rh) until a 0.4 °C fall in rectal temperature, 1 × work cycle**, 10 min rest in climatic chamber	Indoors in a hot factory. Basic level PPE. Intermittent work pattern
4	25	50	20.95	11.54	No	Impermeable	40 min moderate exercise, rest* (~ 22 °C, 50% rh) until a 0.4 °C fall in rectal temperature, 40 min moderate exercise	Indoors, cool conditions. Impermeable PPE
5	40	20	29.45	10.21	No	Permeable	Same as condition 4	Indoors in a hot factory. Basic level PPE
6	40	20	29.45	10.21	No	Impermeable	Same as condition 4	Indoors in a hot factory. Impermeable PPE
7	30	35	28.75	10.68	530 Wm^−2^	Permeable	Same as condition 4	Outdoors on a hot sunny day. Basic level PPE
8	40	20	29.13	10.21	530 Wm^−2^	Permeable	Same as condition 4	Desert environment. Basic level PPE
9	40	35	31.85	17.90	No	Permeable	Same as condition 1	Indoors in a hot factory. Basic level PPE

In conditions 1, 2 and 9, the first period of treadmill walking was extended to 60 min to promote achieving higher rectal temperatures and levels of thermal strain. Conditions 1 and 9 are similar but involve different participants as this study formed part of a larger study aimed to develop models to predict core temperature using non-invasive measures (Richmond et al. 2015)

*PPE *Personal protective clothing, *WBGT *Wet Bulb Globe Temperature, *WVP *Water vapour pressure

ǂ = All work patterns started with a 10 min rest period in the climatic chamber. * = All rest periods were seated. ** = Work cycle—5 min moderate intensity, 5 min moderate-high intensity, 15 min moderate intensity

Due to technical issues, *T*_sk_ was not obtained at the end of one trial in group TTL, and HR was not obtained for two trials in group C and one trial in group TTL. Therefore the n sizes for PSI_original_, PSI_fixed_, aPSI, were 99 (C group) and 34 (TTL group) and for PSI_HS_ 101 (C group) and 34 (TTL group). In the subjective measures (TC and TS), the n sizes were 93 (C group) and 28 (TTL group).

### Physiological parameters

Final *T*_re_, HR and the Δ in *T*_re_ were not significantly different between TTL and C. However, there was a significant difference between TTL and C in: final *T*_sk_; *T*_re_–*T*_sk_; Δ *T*_sk_; ROC in *T*_re_; ROC in *T*_sk_ and ROC in HR; Table 3. The ROC in the last 10 min of a trial was greater in group TTL than group C in the following measures: ROC *T*_re10_; ROC *T*_sk10_; ROC HR_10_; ROC *T*_re10_–*T*_sk10_; Table 3. There were no significant differences in the other physiological parameters measured.

**Table 3:** Mean ± SD of selected measured variables from the participants who completed the trials (Group C) versus those who experienced reaching a thermal tolerance limit (Group TTL)

	*C*	TTL	*p* value	Effect size (*d*)	Effect size (*r*)
Physiological variables					
Final *T*_re_ (°C)	38.57 ± 0.40	38.57 ± 0.44	> 0.05	–	–
Final *T*_sk_ (°C)	37.34 ± 0.88	37.77 ± 0.81	0.014	0.50	–
Final HR (beats min^−1^)	150 ± 20	157 ± 18	> 0.05	–	–
Final *T*_re_–*T*_sk_ (°C)	1.29 ± 0.67	0.80 ± 0.80	0.022	0.60	–
∆ in *T*_re_ (°C)	1.49 ± 0.50	1.46 ± 0.54	> 0.05	–	–
Δ in *T*_sk_ (°C)	4.99 ± 1.14	5.44 ± 1.03	0.034	0.42	–
ROC in *T*_re_ (°C∙min^−1^)	0.013 ± 0.004	0.019 ± 0.010	< 0.001	–	0.27
ROC in *T*_sk_ (°C∙min^−1^)	0.043 ± 0.010	0.073 ± 0.039	< 0.001	–	0.45
ROC in HR (beats min^−1^)	0.66 ± 0.21	0.98 ± 0.49	< 0.001	–	0.31
ROC in *T*_re10_ (°C∙min^−1^)	0.022 ± 0.009	0.032 ± 0.018	< 0.001	–	0.32
ROC in *T*_sk10_ (°C∙min^−1^)	0.019 ± 0.015	0.036 ± 0.022	0.001	–	0.47
ROC in HR_10_ (beats min^−1^)	0.42 ± 0.54	0.82 ± 0.82	0.001	–	0.32
ROC in *T*_re_–*T*_sk10_ (°C∙min^−1^)	0.003 ± 0.010	0.004 ± 0.016	0.015	–	0.22
Physiological strain indices					
PSI_fixed_	6.8 ± 1.5	7.1 ± 1.5	> 0.05	–	–
PSI_original_	6.6 ± 1.6	6.7 ± 1.9	> 0.05	–	–
aPSI	7.6 ± 2.1	8.3 ± 2.2	> 0.05	–	–
PSI_HS_	7.9 ± 1.4	8.4 ± 1.3	> 0.05	–	–
ROC in PSI_fixed_ (min^−1^)	0.06 ± 0.01	0.09 ± 0.04	< 0.001	–	0.45
ROC in PSI_fixed10_ (min^−1^)	0.06 ± 0.03	0.10 ± 0.05	< 0.001	–	0.37
Thermal perceptions					
Final temperature sensation	17.7 ± 1.9	18.8 ± 1.3	0.006	–	0.25
Final thermal comfort	16.3 ± 3.6	18.3 ± 2.0	0.005	–	0.27

Commonly used interpretations of cohen’s *d* and *r* vales is to refer effect sizes as small (*d* ≥ 0.2, *r* = 0.1–0.3), medium (*d* ≥ 0.5, *r* = 0.3–0.5) and large (*d* ≥ 0.8, *r* = 0.5–1.0) (Cohen 1988)

*T*_*re*_ rectal temperature, *T*_*sk*_  skin temperature, *HR *heart rate, *PSI*_*fixed*_ physiological strain index calculated from fixed resting values i.e. 37.0 °C and 70 beats min^−1^, *PSI*_*original*_ physiological strain index calculated from actual resting values, *aPSI *the adaptive physiological strain index, *PSI*_*HS*_  physiological strain index calculated from an estimate of heat storage, *ROC *rate of change

### Physiological strain index

All versions of the PSI assessed (i.e. PSI_fixed_, PSI_HS,_ aPSI) were strongly correlated with PSI_original_ (*r* = 0.869–0.964, *p* < 0.01). Across all conditions, none of the versions of the PSI assessed in the present study differed between TTL and C; Table 3. Due to this outcome, results using PSI_fixed_ will only be described hereafter.

PSI_fixed_ differed across conditions 1, 2 and 6 (*F* (2,44) = 4.28, *p* = 0.020; *ηp*^2^ = 0.163). There was no main effect in PSI_fixed_ for group (*F* (1,44) = 2.55, *p* = 0.117; *ηp*^2^ = 0.055) or an interaction between group and condition (*F* (2,44) = 1.55, *p* = 0.233; *ηp*^2^ = 0.066). However, PSI_fixed_ differed significantly between TTL and C in condition 2 only, but with the final PSI_fixed_ being lower in TTL than C; Table 4.

**Table 4:** Physiological strain index (PSI_fixed_) values of participants who completed the trials (Group C) versus those who reached a thermal tolerance limit (Group TTL) for each of the nine conditions, mean ± SD (range)

Condition	*C*		TTL	
	*n*	PSI_fixed_	*n*	PSI_fixed_
1	11	6.8 ± 1.7 (3.9**–**9.4)	6	6.9 ± 1.8 (4.4**–**8.8)
2	10	7.4 ± 0.9 (6.3**–**9.0)	7	6.1 ± 0.8 (5.3–7.7)*
3	16^†^	5.1 ± 1.4 (2.7**–**6.6)	1	5.2
4	14	6.1 ± 1.0 (4.9**–**7.9)	1	10.5
5	19^†^	6.5 ± 1.4 (4.7**–**9.8)	1	4.7
6	7	8.3 ± 0.9 (7.3**–**9.5)	11^†^	7.6 ± 0.9 (5.8–8.8)
7	14	6.2 ± 1.3	0	–
8	15	7.7 ± 1.0 (6.1**–**10.1)	4	7.7 ± 2.0 (6.0–10.1)
9	9	7.8 ± 1.0 (6.1**–**9.0)	4	7.0 ± 1.1 (6.0–8.0)
All conditions (excl. condition 7)	99	6.8 ± 1.5 (2.7–10.1)*	34	7.1 ± 1.5 (4.4–10.5)

^*^Significantly different PSI_fixed_ between C and TTL groups (*p* = 0.030, *d* = 1.58). The *n* sizes displayed are the actual number of participants who completed or did not complete a trial. Due to no incidences of thermal intolerance occurring in condition 7, this condition was not included in the final analyses. ^†^Due to technical issues, *T*_sk_ was not obtained at the end of one trial in group TTL, and HR was not obtained for two trials in group C and one trial in group TTL. Therefore, in all conditions (excluding condition 7) the n sizes for the final analyses of PSI_fixed_ values were 99 (C group) and 34 (TTL group)

Across all conditions, the ROC in PSI_fixed_ calculated over a trial and over the last 10 min was significantly greater in TTL than C; Table 3. For the ROC in PSI_fixed_ calculated over a trial the main effect of condition was significant (*F* (2,44) = 9.82, *p* < 0.001; *ηp*^2^ = 0.309) as was the main effect for group (*F* (1,44) = 9.65, *p* = 0.003; *ηp*^2^ = 0.309), but not for an interaction between group and condition (*F* (2,44) = 2.37, *p* = 0.105; *ηp*^2^ = 0.097). Within conditions 1, 2 and 6, the ROC in PSI_fixed_ calculated over the exposure was greater in TTL than C in condition 6 only (TTL = 0.12 ± 0.05 min^−1^ vs. *C* = 0.07 ± 0.01 min^−1^, *p* = 0.001, *d* = 1.15).

The ROC in PSI_fixed_ over the last 10 min (ROC_fixed10_) showed a main effect of condition (*F* (2,44) = 12.15, *p* < 0.001; *ηp*^2^ = 0.361), but there was no main effect for group (*F* (1,44) = 0.82, *p* = 0.771; *ηp*^2^ = 0.002) nor an interaction between group and condition (*F* (2,44) = 0.25, *p* = 0.771; *ηp*^2^ = 0.012).

**Fig. 2:**
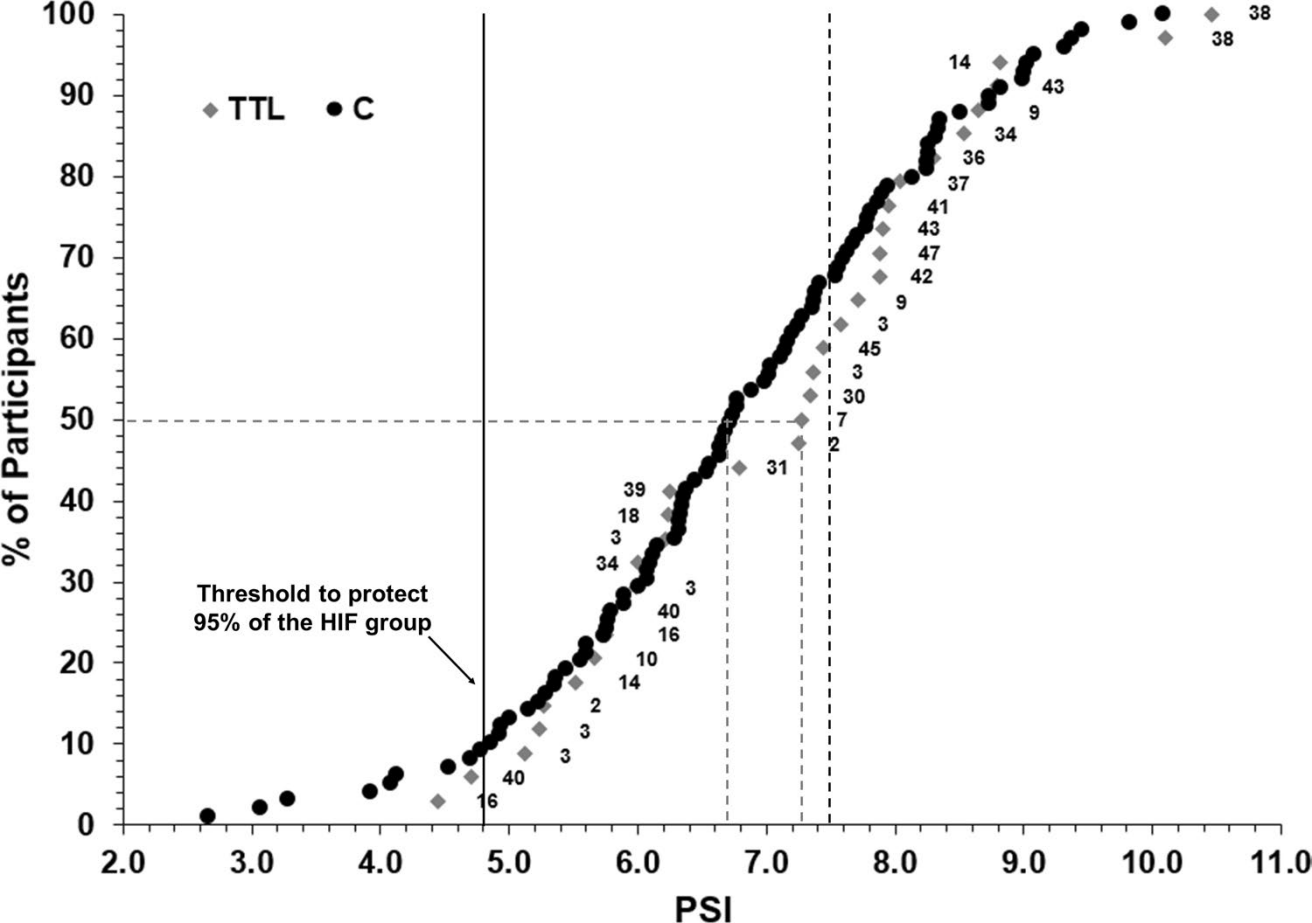
Cumulative frequency showing % of participants dropping out at each Physiological Strain Index (PSI_fixed_) value for group TTL and group C. The grey dashed line shows the PSI_fixed_ value when 50% of participants dropped out or completed the trial. The black dashed line represents Buller’s (2008) ‘at risk’ classification of 7.5. The black solid line represents the threshold in PSI_fixed_ required to protect 95% of the TTL group. The numbers associated to the cases in group TTL is the participant identifier corresponding to that case

Figure 2 is a cumulative frequency graph displaying the final PSI_fixed_ between groups TTL and C. In Fig. 2 some of the PSI_fixed_ values are over 10 because HR was higher than the estimated maximum HR (180 beats min^−1^) included in the PSI_fixed_ equation. In group TTL, 58% of cases had a PSI < 7.5. In addition, 29.4% did not reach a PSI_fixed_ over 6 (the ‘high’ PSI zone) when they stopped prematurely due to reaching a thermal tolerance limit. The grey dashed lines show the PSI_fixed_ when 50% of the individuals stopped prematurely (PSI_fixed_ = 7.3) or completed a trial (PSI_fixed_ = 6.7). Figure 2 demonstrates that to protect 95% of individuals from reaching a thermal tolerance limit in the TTL group, they would need to stop at a PSI_fixed_ value of 4.8. However, a termination criteria of a PSI_fixed_ of 4.8 would result in 91.0% of the trials being stopped prematurely in group C, which otherwise would have been completed safely. Figure 2 also shows the wide inter- and intra-individual variability of the final PSI_fixed_ in group TTL. This variability illustrates that there is no clear PSI_fixed_ value (or range of PSI_fixed_ values) that distinguishes the two groups (i.e. group TTL and group C) or is associated with reaching a thermal tolerance limit within an individual. For example, participant 2 reaches a thermal tolerance limit at a PSI_fixed_ value of 7.2 in one condition, but reaches a thermal tolerance limit at a PSI_fixed_ value of 5.3 in another condition.

**Fig. 3:**
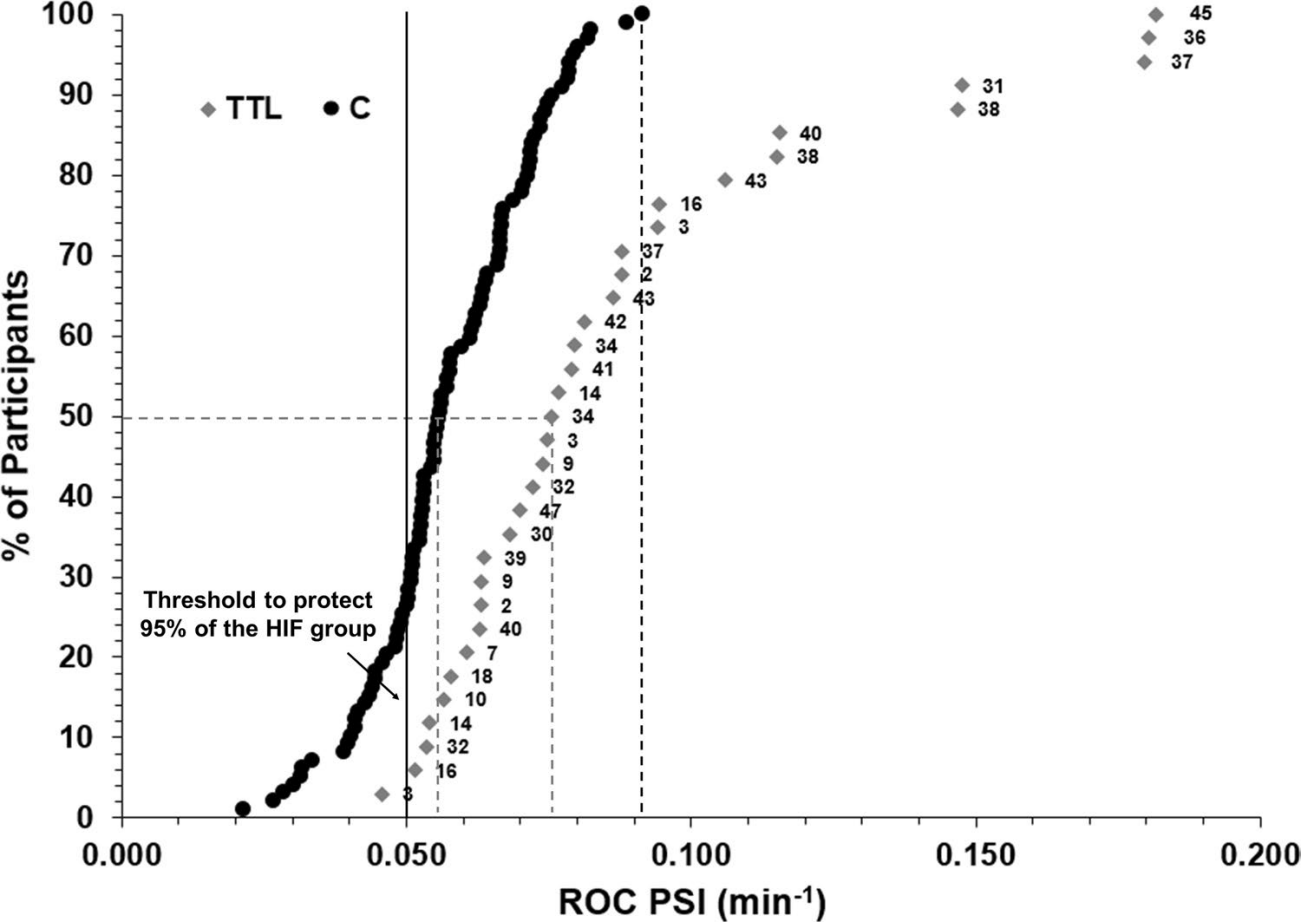
Cumulative frequency showing % of participants dropping out at each rate of change (ROC) in the Physiological Strain Index (PSI_fixed_) for group TTL and group C. The grey dashed line shows the ROC in PSI_fixed_ when 50% of participants dropped out or completed the trial. The black dashed line represents the upper limit in ROC in PSI_fixed_ to complete a trial. The black solid line represents the threshold in PSI_fixed_ required to protect 95% of the TTL group. The numbers associated to the cases in group TTL is the participant identifier corresponding to that case

Figure 3 illustrates that if ROC in PSI_fixed_ is used instead, to protect 95% of individuals from reaching a thermal tolerance limit in the TTL group, they would need to stop when their ROC in PSI_fixed_ exceeds a value of 0.05 min^−1^. This would result in 79.0% of the trials being stopped prematurely in the C group which otherwise would have been successfully completed. In addition, 29.4% of the participants in group TTL had a ROC in PSI_fixed_ above 0.09 min^−1^, which is the highest value obtained in the C group.

### Perceptual parameters.

There was a significant difference between TTL and C in both TS and TC; Table 3.

## Discussion

Based on previous literature it was recognised that PSI has the potential to identify individuals at risk of HIF and/or other heat-related symptoms (Buller et al. 2008). In the present study, participants reaching a thermal tolerance limit started to occur around a PSI_fixed_ value of 4.5, above which the occurrence increased almost linearly with PSI_fixed_ before maximising around a PSI_fixed_ value of 9. However, Fig. 2 clearly illustrates that PSI_fixed_ does not discriminate between participants who were able to complete an exercise bout in thermally stressful conditions versus those who were unable to due to reaching a thermal tolerance limit. Moreover, in condition 2, opposite to the expectation, the mean PSI_fixed_ value was lower in the TTL group than group C, reinforcing the inability of PSI_fixed_ to identify individuals at risk of reaching a thermal tolerance limit. Furthermore, bearing in mind that according to Buller et al. (2008) and Moran et al. (1998b) a PSI value above 7.5 is considered ‘at risk’ from thermal injury, 58% of the TTL group experienced HIF or other symptoms of heat-related illnesses causing them to stop exercising before reaching this suggested ‘at risk’ limit value and 29.4% did not even reach a PSI_fixed_ value of 6 (considered the ‘high’ PSI zone). This highlights that if a value of 6 on the PSI_fixed_ was used to identify people at risk of reaching a thermal tolerance limit, ~ 29% of the heat exposures where participants reached a thermal tolerance limit would not have been detected on time, posing a health and safety risk. The results from this study indicate that to protect 95% of the population from HIF or a heat-related illness, a PSI_fixed_ of 4.8 could be used, but this may be considered too conservative as it could result in stopping the majority of workers (~ 91%) successfully completing certain physical tasks. To overcome this problem, it has been identified that monitoring the rate of change in PSI_fixed_ may be more appropriate to identify individuals at risk of reaching a thermal tolerance limit, rather than utilising an absolute PSI_fixed_ value. The use of other physiological and perceptual measures such as skin temperature or temperature sensation may also be beneficial.

As aforementioned, the notion of a ‘critical’ core temperature has been shown to be less predictive of HIF than other factors, such as heart rate, skin temperature and the gradient between *T*_c_ and *T*_sk_ (Cuddy et al. 2014; Ely et al. 2009; Pandolf and Goldman 1978; Périard et al. 2011; Schlader et al. 2011). The present study provides further evidence with final *T*_re_ being similar between the two groups (i.e. ~ 38.5 °C). In addition, similarily to previous observations (Montain et al. 1994), some participants in the present study reached a thermal tolerance limit at a far lower *T*_re_ than ‘critical’ core temperatures of 39.0–40.0 °C, i.e. 37.9–38.0 °C. This may explain why the PSI_fixed_ equation underestimated the physiological strain experienced in the majority of cases in the TTL group as the PSI_fixed_ includes an assumed ‘critical’ core temperature of 39.5 °C. This observation supports the incorporation of a core temperature of 38.0 °C as a good safety margin in many occupational heat stress guidelines to protect the majority of the population of developing a heat-related illness (ACGIH, 2009; ISO 7933 2004). However, as previously highlighted, a core temperature limit of 38.0 °C may be considered too conservative in certain scenarios mainly due to core temperatures associated with thermal tolerance being influenced by several factors such as aerobic training, acclimation status, hydration status, exercise intensity, clothing and environmental parameters (e.g. hot-dry vs hot-wet environments) (Cheung and McLellan 1998; Montain et al. 1999; Stewart et al. 2014). The present study suggests that monitoring the rate of change in core temperature over a set period, such as ten minutes, may provide a better predictor of reaching of a thermal tolerance limit than a specific absolute core temperature.

The present study provides further evidence that a higher skin temperature and the smaller gradient between *T*_c_ and *T*_sk_ is associated with a thermal tolerance limit as both parameters were different between groups TTL and C at the end of a trial (i.e. a difference of ~ 0.43 °C in *T*_sk_ and ~ 0.49 °C in the gradient between *T*_c_ and *T*_sk_). Several studies have identified skin temperature as a modulator for exercise intensity (Schlader et al. 2011; Schulze et al. 2015) and ratings of perceived exertion (RPE) (Armada-da-Silva et al. 2004) whilst exercising in the heat. In addition, when encapsulating/impermeable PPE is worn, reduced work tolerance times generally correspond with lower core temperatures and higher skin temperatures compared to when non-encapsulating/permeable PPE is worn (McLellan and Havenith 2016; Montain et al. 1994; Stewart et al. 2014). This evidence suggests incorporating skin temperature into heat strain indices/heat stress monitors may improve the validity of identifying individuals at risk of reaching a thermal tolerance limit in a wide range of thermally stressful conditions.

To provide more accurate reflections of physiological strain in scenarios where HIF may occur at core temperatures below 39.5 °C, Buller et al. (2016) developed the adaptive PSI (aPSI). As previously described, the aPSI adjusts the critical core temperature of 39.5 °C used in the original PSI by the gradient between *T*_c_ and *T*_sk_. Even though this modified version has been demonstrated to better identify levels of heat strain than the original PSI (Buller et al. 2016), with larger *T*_c_–*T*_sk_ gradients resulting in a lower PSI, the present study demonstrates that it is unable to identify individuals reaching a thermal tolerance limit in a wide range of thermally stressful conditions and therefore caution must be adopted when utilised in thermally stressful occupations.

Even though absolute *T*_re_ was not found to be associated with reaching a thermal intolerance limit, the rate of heat storage (reflected in the ROC in *T*_re_ and *T*_sk_) was different between TTL and C over a whole trial and in the last 10 min of a trial. The rate at which heat is stored within the body has previously been shown to cause an alteration in exercise intensity (Tucker et al. 2006) possibly due to the anticipatory regulation of exercise intensity which is eloquently described in Marino, 2004 and Tatterson et al. 2000. This may explain why the rate of change in PSI_fixed_ observed in the current study was different between TTL and C, but absolute PSI_fixed_ was not. The absolute rise in both *T*_re_ and HR did not differ between the two groups, i.e. ~ 1.45 °C and 77 beats min^−1^, respectively, but the rate of change in *T*_re_ and HR were both different. In the study by Tucker et al. (2006) the difference in the rate of rise in heat storage between the condition that caused a greater reduction in exercise intensity was mainly driven by the rate of change in mean skin temperature. This observation reinforces the importance of skin temperature as a determinant of TTL and may explain why all the parameters associated with skin temperature, i.e. final *T*_sk_, ROC in *T*_sk_ and *T*_sk10_ and the absolute change in *T*_sk_, did discriminate between the two groups. However, when skin temperature in the form of heat storage is incorporated into the PSI equation (i.e. PSI_HS_), it does not sufficiently improve its ability to identify individuals reaching a thermal tolerance limit.

Temperature sensation and thermal comfort have also been highlighted as key regulators of exercise intensity, especially at lower levels of hyperthermia (Flouris and Schlader 2015). To increase the practicality of indices that estimate physiological strain, heat strain indices using only perceptual measures have been developed (Borg et al. 2017; Gallagher et al. 2012; Tikuisis et al. 2002). For example, Tikuisis et al. (2002) developed a perception-based version of the PSI, (i.e. PeSI), replacing heart rate and core temperature with temperature sensation and RPE and using the upper limits of the perceptual scales (13 = intolerably hot and 10 = maximal exertion) as critical values. The perception-based heat strain index was validated against the PSI to assess physiological strain in aerobically trained and untrained participants performing open-ended moderate exercise in a hot-dry environment (40 °C, 30% rh) while wearing semipermeable protective clothing. The untrained participants ceased exercise sooner than the trained participants (69 vs 95 min) with a lower mean core temperature (38.58 °C vs 39.21 °C) and PSI value (6.7 vs. 8.2), but with a similar mean heart rate (~ 163 beats min^−1^). However, both groups ceased exercise with a similar rating of PeSI (~ 6.5) suggesting that PeSI is a better predictor of an individual’s TTL than PSI. In the present study, the participants in the TTL group reported feeling hotter and more uncomfortable compared to participants who completed a trial, even though core temperature was similar between the two groups. However, the difference in skin temperature observed between the two groups might be driving this difference in thermal perception. Unfortunately, RPE was not measured in all trials in the present study, therefore, we were unable to assess the ability of PeSI to identify risk of reaching a thermal tolerance limit.

In regard to both validity and practicality, perceptual-based indices similar to the PeSI may be more appropriate to use than PSI to identify individuals at risk of reaching a thermal tolerance limit. However, as highlighted in Tikuisis et al. (2002), in some individuals who are highly motivated, or aerobically trained, there is the potential for them to underestimate their physiological strain which places them at risk of a thermal injury such as heat exhaustion or heat stroke. The inclusion of a physiological parameter into a perceptual-based index may counteract this. The results from the present and previous studies, such as Cuddy et al. (2013), suggest that the inclusion of skin temperature or heart rate, especially the rate of change in these two measures, may offer a plausible solution. In both solutions, including a rate of change of a physiological parameter over the previous ten minutes, rather than over a whole exposure, would provide a better depiction of the thermal state of the individual. This is important as work scenarios tend to involve metabolic activity that is intermittent in nature and can last several hours.

Due to the multi-factorial nature of HIF, it could be considered understandable that including a combination of perceptual/physiological and psychological parameters would increase the probability of correctly identifying individuals at risk of reaching a thermal tolerance limit. However, one drawback of increasing the number of parameters included in a heat strain index/heat stress monitor is the risk of reducing their usability in work-place settings as they may become impractical and/or too expensive. In regard to producing an index that protects the majority of people without compromising productivity, another difficulty for all proposed indices will be establishing the critical threshold in any parameters used. Thresholds are likely to be highly individualised and influenced by the interplay between changes in skin temperature and core temperature and their effect on the cardiovascular, respiratory and central nervous systems.

## Limitations

While the data collected for this study provide strong evidence regarding the limited utility of PSI as a protective index, they unfortunately do not allow a more detailed analysis of threshold limit values. If all participants had exercised to exhaustion (or voluntary cessation) in all conditions, it would be easier to identify specific thresholds conducive for individuals reaching a thermal tolerance limit in the physiological and perceptual parameters measured. However, such an experiment series would have been extremely stressful for participants and repeating such exhaustive trials many times may have been difficult to do reliably and safely.

## Conclusion

In summary, the findings from the present study suggest that the absolute PSI is not a valid measure to identify workers at risk from HIF and/or other heat related symptoms associated with a thermal tolerance limit and caution should be taken if utilised within thermally stressful occupations. This is the case for all variations of PSI considered: PSI_original_, PSI_fixed_, aPSI and PSI_HS_. Similarly absolute *T*_re_ or HR were not predictive of reaching a thermal tolerance limit. However, there is potential for the rate of change in PSI or a physiological-perceptual strain index that incorporates a combination of either a rate of change in *T*_re_, *T*_sk_ or HR with thermal perceptions to be a more valid measure. Further investigations are required to validate these suggested changes to PSI.

## Abbreviations

HIF: Hyperthermia-induced fatigue
TTL: Thermal tolerance limit
PSI: Physiological strain index
PSI_original_: The physiological strain index calculated from actual resting values
PSI_fixed_: The physiological strain index calculated from fixed resting values i.e. 37.0 °C and 70 b min^−^^1^
aPSI: The adaptive physiological strain index
PSI_HS_: The physiological strain index calculated from an estimate of heat storage
*T*_c_: Core temperature
*T*_sk_: Skin temperature
*T*_re_: Rectal temperature
*T*_re_–*T*_sk_: The gradient between *T*_re_ and *T*_sk_
*T*_re10_: Rate of change in rectal temperature over the last 10 minutes of a trial
*T*_sk10_,: Rate of change in skin temperature over the last 10 minutes of a trial
HR_10_,: Rate of change in heart rate over the last 10 min of a trial
*T*_re10_–*T*_sk10_: Rate of change in the gradient between *T*_re_ and *T*_sk_ over the last 10 minutes of a trial
PSI_fixed10_: Rate of change in PSI_fixed_ over the last 10 minutes of a trial
HR: Heart rate
PPE: Personal protective equipment
TC: Thermal comfort
TS: Temperature sensation
ROC: Rate of change
$\dot{V}O_{{2{\text{peak}}}}$: Peak oxygen uptake
